# Association of Serum Lactate Dehydrogenase With Sepsis‐Associated Acute Kidney Injury: A Large‐Scale Analysis Based on the MIMIC‐IV Database

**DOI:** 10.1155/emmi/9179449

**Published:** 2026-08-24

**Authors:** Nu Zhang, Zhiyu Wu, Mengyuan Luo, Zhihua Mi, Qiqing Shi

**Affiliations:** ^1^ Department of Anesthesiology, Minhang Hospital, Fudan University, Shanghai 201199, China, fudan.edu.cn; ^2^ Department of Anesthesiology and Perioperative Medicine, Shanghai Fourth People’s Hospital, School of Medicine, Tongji University, Shanghai 200434, China, tongji.edu.cn

**Keywords:** biomarker, critical care, lactate dehydrogenase, MIMIC-IV database, sepsis-associated acute kidney injury

## Abstract

**Background:**

Sepsis‐associated acute kidney injury (SA‐AKI) frequently occurs during critical illness and represents a serious complication of sepsis. Although serum lactate dehydrogenase (LDH) may reflect tissue injury and metabolic derangement, large‐cohort evidence relating LDH to SA‐AKI remains limited.

**Methods:**

We analyzed a retrospective cohort of adults with sepsis identified in Medical Information Mart for Intensive Care IV (MIMIC‐IV). The exposure was serum LDH recorded during the first 24 h after ICU admission, grouped by quartile. Associations with SA‐AKI were estimated using logistic regression models with stepwise covariate adjustment for demographics, comorbid disease, illness severity, and selected metabolic and inflammatory laboratory variables. Restricted cubic splines were fitted to assess possible nonlinearity.

**Results:**

The final cohort comprised 9461 patients. The incidence of SA‐AKI increased from 44.2% in the lowest LDH quartile to 70.6% in the highest quartile (*p* < 0.001). In the expanded model including demographics, comorbidities, illness severity, lactate, white blood cell count, and glucose, higher LDH levels retained a statistically significant association with SA‐AKI (adjusted OR: 1.033 per 100 U/L, 95% CI: 1.025–1.042, *p* < 0.001). Spline modeling indicated nonlinearity, with the curve becoming steeper in the higher LDH range.

**Conclusion:**

In this MIMIC‐IV cohort, LDH values obtained during the first 24 h after ICU admission were positively associated with SA‐AKI. Because the analysis was observational, the results should be viewed as associative rather than causal.

## 1. Introduction

Sepsis‐associated acute kidney injury (SA‐AKI) is frequently observed in critically ill patients and contributes substantially to morbidity and mortality [1, 2]. The development of SA‐AKI is multifactorial, involving circulatory disturbances, maladaptive inflammatory responses, and major changes in cellular metabolism [3, 4]. Recent work has increasingly placed metabolic dysfunction at the center of septic kidney injury. In renal tubular epithelial cells, sepsis may shift energy production away from mitochondrial oxidative phosphorylation toward aerobic glycolysis, a Warburg‐like pattern that has been implicated in renal injury during sepsis [5–7].

Lactate dehydrogenase (LDH) is a glycolytic enzyme that interconverts pyruvate and lactate with concurrent NADH/NAD^+^ cycling, thereby linking glycolytic flux to cellular redox balance and energy metabolism [8]. In clinical settings, elevated circulating LDH is commonly viewed as a nonspecific marker of tissue injury, inflammatory activation, hypoxia, enhanced glycolytic metabolism, and broader metabolic stress in sepsis and has been associated with unfavorable outcomes [9, 10]. Experimental data also suggest that LDH‐related lactate accumulation and lactylation of proteins involved in inflammation, mitochondrial dysfunction, and tubular epithelial injury could provide biological context for a link between metabolic dysregulation and SA‐AKI [11–13].

Although prior work supports the relevance of LDH in sepsis and in AKI‐related conditions, evidence focused specifically on serum LDH and SA‐AKI in large clinical cohorts remains limited. We, therefore, examined whether serum LDH measured within 24 h after ICU admission was associated with SA‐AKI among adult septic patients in the Medical Information Mart for Intensive Care IV (MIMIC‐IV).

## 2. Methods

### 2.1. Data Source

Data were obtained from MIMIC‐IV (Version 3.1), and reporting followed the Strengthening the Reporting of Observational Studies in Epidemiology (STROBE) guidance for cohort studies. MIMIC‐IV was created through a collaboration between the Massachusetts Institute of Technology and Beth Israel Deaconess Medical Center and includes hospital data from admissions between 2008 and 2022. Available data elements include patient demographics, vital signs, laboratory tests, medication information, diagnosis codes, and outcome records.

Patient identifiers have been removed from the MIMIC‐IV dataset. The investigators accessed the database after completing the required training and obtaining PhysioNet authorization (Record ID: 71467776). Because only deidentified data were analyzed, no additional institutional ethics approval was required for this secondary analysis.

### 2.2. Study Population

We performed a retrospective cohort analysis using MIMIC‐IV. Eligible adults with sepsis were identified from their first recorded ICU stay. Sepsis‐3 criteria were used, requiring suspected or documented infection together with a SOFA score of at least 2. Within MIMIC‐IV, suspected infection was identified from paired microbiological culture testing and antibiotic administration within the accepted Sepsis‐3 time window; SOFA was calculated from variables available during the first 24 h after ICU admission.

Patients were included when they met all of the following conditions: (1) sepsis according to Sepsis‐3; (2) age of 18 years or older; (3) first ICU stay in the database; (4) ICU stay of at least 48 h; and (5) available serum LDH, urine output, and renal function data during the first 24 h after ICU admission.

We excluded patients with (1) chronic dialysis or end‐stage renal disease; (2) insufficient serum creatinine or urine output data for SA‐AKI ascertainment; (3) acute myocardial infarction or acute hepatitis/hepatic necrosis within 24 h after ICU admission, because these conditions may cause non‐renal LDH elevation [14, 15]; or (4) no serum LDH measurement during the first 24 h after ICU admission.

### 2.3. Definition of Acute Kidney Injury

AKI ascertainment followed the 2012 Kidney Disease: Improving Global Outcomes (KDIGO) criteria [16], using any of the following: serum creatinine reaching at least 1.5 times baseline within 7 days, serum creatinine rising by at least 0.3 mg/dL within 48 h, or urine output below 0.5 mL/kg/h for more than 6 h. For AKI ascertainment, baseline creatinine was taken as the earliest available creatinine value within the assessment window. For patients with repeated creatinine tests, later values were compared with this baseline according to KDIGO criteria. SA‐AKI denoted KDIGO‐defined AKI in patients meeting Sepsis‐3 criteria.

### 2.4. Variable Extraction and Definitions

Extracted demographic variables were age, gender, body mass index (BMI), and race. Laboratory data from the first 24 h included renal indices (serum creatinine and urine output), metabolic variables (lactate and glucose), and white blood cell (WBC) count as an inflammatory indicator. Comorbidities and severity measures included hypertension (HTN), diabetes mellitus (DM), chronic obstructive pulmonary disease (COPD), and the SOFA score during the first 24 h after ICU admission. The primary outcome was SA‐AKI. Continuous renal replacement therapy (CRRT), 28‐day ICU mortality, and 28‐day in‐hospital mortality were summarized as descriptive clinical outcomes.

### 2.5. Statistical Analysis

Analyses were performed in DecisionChain software (Version 1.0; Hangzhou Yuantong Information Technology Co., Ltd., Hangzhou, China). Statistical significance was defined using a two‐sided *p* value below 0.05.

Descriptive statistics: Continuous variables are reported as medians and interquartile ranges (IQRs). Between‐group comparisons used the Kruskal–Wallis test. Categorical data were evaluated with the chi‐square test or Fisher’s exact test, as appropriate.

Multivariable logistic regression: LDH was entered into logistic regression models both as quartiles (Q1–Q4) and as a continuous exposure to estimate its association with SA‐AKI. Model 1 included no covariates. Model 2 adjusted for age, gender, BMI, and race. Model 3 further adjusted for SOFA score and comorbidities, including HTN, diabetes, and COPD. The expanded Model 4 additionally included lactate, WBC count, and glucose to account for metabolic and inflammatory status. Serum creatinine and urine output were not included as adjustment covariates because they are components of KDIGO‐based SA‐AKI ascertainment and could introduce overadjustment. Covariates were selected on clinical grounds and with reference to a directed acyclic graph describing assumed links among demographics, comorbidities, illness severity, metabolic/inflammatory status, LDH, and SA‐AKI (Supporting Figure S1).

Missing data handling: Patients were not entered into the primary analytic cohort if serum LDH was unavailable or if the information needed to ascertain SA‐AKI was incomplete. To evaluate possible selection bias from these exclusions, we compared baseline characteristics of included patients with those excluded for missing LDH measurements or incomplete SA‐AKI ascertainment data. Because some key variables were unavailable in the excluded group, descriptive comparisons used available observations, with variable‐specific sample sizes reported where relevant.

Among candidate covariates for multivariable modeling, variables missing in more than 25% of the patients were not included in adjusted models, whereas variables with missingness of 25% or less were handled using the multiple imputation module in DecisionChain. The imputation model incorporated the exposure variable, the outcome variable, and all covariates used for adjustment in the multivariable models. Supporting Table S1 lists preimputation missingness for candidate covariates. To examine the robustness of the missing‐data approach, we repeated Models 1–4 in a complete‐case cohort restricted to patients with complete information for every covariate in the expanded Model 4.

Sensitivity analysis for temporal overlap: We addressed potential overlap between LDH assessment and SA‐AKI ascertainment by repeating the analysis after removing patients who met KDIGO criteria for AKI during the first 24 h after ICU admission. Patients with AKI but no available timing information were also removed from this sensitivity analysis. Here, delayed SA‐AKI referred to AKI first identified after 24 h and within 7 days after ICU admission. The same logistic regression framework was then applied to delayed SA‐AKI.

Restricted cubic spline (RCS) analysis: RCS models were fitted to assess whether the LDH–SA‐AKI association departed from linearity. Spline knots were specified at the 20th, 40th, 60th, and 80th percentiles of the LDH distribution.

Subgroup and interaction analyses: We performed stratified analyses by age (< 60 vs. ≥ 60 years), gender, BMI (< 24 vs. ≥ 24 kg/m^2^), race, HTN, DM, and COPD. Interaction terms between LDH and subgroup variables were added to the regression model.

## 3. Results

### 3.1. Baseline Characteristics of the Study Population

**FIGURE 1:**
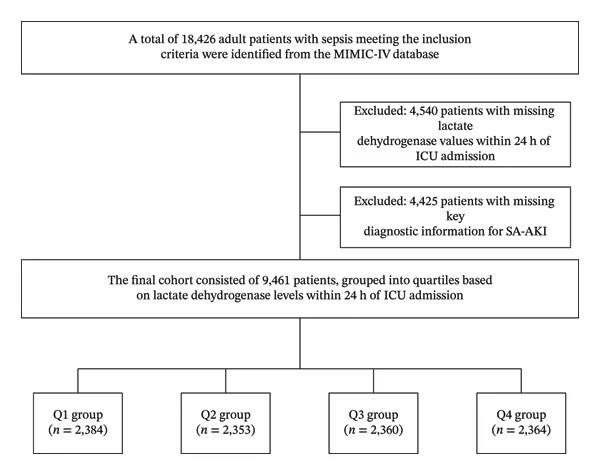
Flowchart of cohort selection. From 18,426 adult septic patients identified in MIMIC‐IV, 4540 were removed because LDH was not measured within 24 h after ICU admission and 4425 were removed because key SA‐AKI diagnostic information was unavailable. The final cohort included 9461 patients, who were grouped by LDH quartile (Q1–Q4).

A total of 9461 adult patients with sepsis meeting the Sepsis‐3 criteria were included from the MIMIC‐IV database (Figure 1). Patients were stratified into four groups according to the quartiles of serum LDH levels measured within 24 h of ICU admission: Q1 (≤ 229 U/L, *n* = 2384), Q2 (230–313 U/L, *n* = 2353), Q3 (314–478 U/L, *n* = 2360), and Q4 (> 478 U/L, *n* = 2364).

Baseline demographic and clinical characteristics are summarized in Table 1. The median age of the cohort was 66 years (IQR: 55–78), and 58.3% of the patients were male. Higher LDH levels were associated with more severe illness and worse laboratory profiles. Specifically, the SOFA score increased progressively from 6.0 (IQR: 3.5–8.0) in Q1 to 8.0 (IQR: 5.0–11.0) in Q4 (*p* < 0.001). Renal function markers such as creatinine, as well as inflammatory markers including WBC count, also increased significantly across LDH quartiles (all *p* < 0.001). Metabolic parameters showed a similar pattern: lactate levels rose from 1.70 mmol/L in Q1 to 2.40 mmol/L in Q4, and glucose levels increased from 128.0 mg/dL to 144.0 mg/dL, indicating greater metabolic stress. The distribution of comorbidities differed slightly across groups. The proportion of HTN decreased from 40.6% in Q1 to 34.3% in Q4, and COPD also showed a modest decline, while diabetes prevalence remained relatively stable.

**TABLE 1:** Comparison of baseline clinical and laboratory characteristics among LDH quartiles in patients with sepsis.

Exposure (LDH)	Q1 *N* = 2384	Q2 *N* = 2353	Q3 *N* = 2360	Q4 *N* = 2364	*p* value
Lactate, median (p25–p75)	1.70 (1.10–2.58)	1.90 (1.30–2.60)	1.90 (1.30–2.90)	2.40 (1.60–4.20)	< 0.001
Age, median (p25–p75)	67.00 (56.00–78.00)	68.00 (57.00–79.00)	66.00 (55.00–77.00)	63.00 (52.00–74.00)	< 0.001
BMI, median (p25–p75)	27.18 (23.37–31.61)	27.79 (24.11–33.13)	28.25 (24.26–33.47)	28.51 (24.78–33.49)	< 0.001
Gender, *n* (%)					0.104
F	980 (41.11%)	975 (41.44%)	1035 (43.86%)	959 (40.57%)	
M	1404 (58.89%)	1378 (58.56%)	1325 (56.14%)	1405 (59.43%)	
Race, *n* (%)					< 0.001
White	1542 (64.68%)	1445 (61.41%)	1387 (58.77%)	1287 (54.44%)	
Black	160 (6.71%)	146 (6.20%)	198 (8.39%)	213 (9.01%)	
Other	682 (28.61%)	762 (32.38%)	775 (32.84%)	864 (36.55%)	
SOFA, median (p25–p75)	6.00 (3.50–8.00)	6.00 (4.00–9.00)	7.00 (4.00–10.00)	8.00 (5.00–11.00)	< 0.001
Creatinine, median (p25–p75)	1.00 (0.70–1.70)	1.10 (0.80–1.70)	1.10 (0.80–1.85)	1.30 (0.90–2.20)	< 0.001
White blood cells, median (p25–p75)	11.10 (7.70–15.40)	11.80 (8.30–16.50)	12.70 (8.60–18.30)	13.40 (9.00–18.60)	< 0.001
Glucose, median (p25–p75)	128.00 (103.00–165.50)	135.00 (111.00–174.00)	134.50 (109.00–178.00)	144.00 (114.00–201.50)	< 0.001
HTN, *n* (%)					< 0.001
No	1416 (59.40%)	1418 (60.26%)	1514 (64.15%)	1552 (65.65%)	
Yes	968 (40.60%)	935 (39.74%)	846 (35.85%)	812 (34.35%)	
DM, *n* (%)					< 0.001
No	1651 (69.25%)	1592 (67.66%)	1640 (69.49%)	1680 (71.07%)	
Yes	733 (30.75%)	761 (32.34%)	720 (30.51%)	684 (28.93%)	
COPD, *n* (%)					< 0.001
No	1940 (81.38%)	1951 (82.92%)	1971 (83.52%)	2032 (85.96%)	
Yes	444 (18.62%)	402 (17.08%)	389 (16.48%)	332 (14.04%)	
SA‐AKI, *n* (%)					< 0.001
No	1331 (55.83%)	1149 (48.83%)	970 (41.10%)	694 (29.36%)	
Yes	1053 (44.17%)	1204 (51.17%)	1390 (58.90%)	1670 (70.64%)	
CRRT, *n* (%)					< 0.001
No	2192 (91.95%)	2111 (89.72%)	2030 (86.02%)	1714 (72.50%)	
Yes	192 (8.05%)	242 (10.28%)	330 (13.98%)	650 (27.50%)	
Death within ICU 28 days, *n* (%)					< 0.001
No	2023 (84.86%)	1904 (80.92%)	1811 (76.74%)	1578 (66.75%)	
Yes	361 (15.14%)	449 (19.08%)	549 (23.26%)	786 (33.25%)	
Death within hospital 28 days, *n* (%)					< 0.001
No	2045 (85.78%)	1931 (82.07%)	1834 (77.71%)	1603 (67.81%)	
Yes	339 (14.22%)	422 (17.93%)	526 (22.29%)	761 (32.19%)	

*Note:* Values are medians (IQRs) or *n* (%). Groups were compared using the Kruskal–Wallis test or chi‐square test.

Abbreviations: BMI, body mass index; COPD, chronic obstructive pulmonary disease; CRRT, continuous renal replacement therapy; DM, diabetes mellitus; HTN, hypertension; LDH, lactate dehydrogenase; SA‐AKI, sepsis‐associated acute kidney injury; SOFA, sequential organ failure assessment.

Regarding clinical outcomes, the incidence of SA‐AKI increased markedly with LDH elevation: 44.2% in Q1, 51.2% in Q2, 58.9% in Q3, and 70.6% in Q4 (*p* < 0.001). Similarly, the proportion of patients requiring CRRT rose from 8.1% in Q1 to 27.5% in Q4. Both ICU 28‐day mortality and in‐hospital 28‐day mortality increased significantly across LDH quartiles (*p* < 0.001). Overall, higher LDH levels were closely associated with greater disease severity, heightened inflammatory response, metabolic disturbance, and poorer clinical outcomes. These additional outcomes were analyzed descriptively across LDH quartiles and should not be interpreted as evidence of formal prognostic utility.

To assess potential selection bias, we compared baseline characteristics between included and excluded patients. As shown in Supporting Table S2, included patients were younger but had higher SOFA scores, longer ICU and hospital stays, and higher 28‐day ICU and in‐hospital mortality. BMI and diabetes prevalence were similar between groups, whereas sex distribution, race, HTN, and COPD differed slightly but significantly. These differences suggest that the final analytic cohort may represent patients with greater illness severity and more complete clinical monitoring; therefore, selection bias related to missing data cannot be fully excluded.

### 3.2. Multivariable Logistic Regression Analysis

Multivariable logistic regression demonstrated a significant positive association between LDH levels and the risk of SA‐AKI (Table 2). When LDH was modeled as a continuous variable, higher LDH levels were consistently associated with increased odds of SA‐AKI across Models 1–3. For each 100 U/L increase in LDH, the ORs were 1.048 (95% CI: 1.040–1.058) in Model 1, 1.052 (95% CI: 1.043–1.062) in Model 2, and 1.036 (95% CI: 1.028–1.045) in Model 3 (all *p* < 0.001). These associations remained statistically significant after adjustment for demographic characteristics, comorbidities, and SOFA score.

**TABLE 2:** Association between lactate dehydrogenase (LDH) levels and the risk of SA‐AKI in multivariable logistic regression models.

Exposure (LDH)	Model 1 OR (95% CI)	Model 2 OR (95% CI)	Model 3 OR (95% CI)	Model 4 OR(95% CI)
Continuous variable (per 100 U/L increase)	1.048 (1.040–1.058)^∗∗∗^	1.052 (1.043–1.062)^∗∗∗^	1.036 (1.028–1.045)^∗∗∗^	1.033 (1.025–1.042)^∗∗∗^
Categorical variable (quartile)				
Q1 group (reference)	1	1	1	1
Q2 group	1.324 (1.182–1.485)^∗∗∗^	1.278 (1.137–1.435)^∗∗∗^	1.203 (1.064–1.361)^∗∗∗^	1.194 (1.055–1.350)^∗∗^
Q3 group	1.811 (1.614–2.033)^∗∗∗^	1.791 (1.593–2.015)^∗∗∗^	1.602 (1.416–1.814)^∗∗∗^	1.585 (1.399–1.795)^∗∗∗^
Q4 group	3.042 (2.699–3.430)^∗∗∗^	3.194 (2.821–3.611)^∗∗∗^	2.559 (2.247–2.916)^∗∗∗^	2.466 (2.158–2.816)^∗∗∗^

*Note:* Model 1: no covariate adjustment. Model 2: included age, gender, BMI, and race. Model 3: additionally included SOFA score and comorbidities (hypertension, diabetes, and COPD). Model 4: additionally included lactate, white blood cell count, and glucose. Continuous LDH was scaled per 100 U/L. LDH quartiles were Q1 ≤ 229 U/L, Q2 230–313 U/L, Q3 314–478 U/L, and Q4 > 478 U/L. ^∗^ *p* < 0.05; ^∗∗^ *p* < 0.01; and ^∗∗∗^ *p* < 0.001.

When LDH was analyzed by quartiles, the risk of SA‐AKI increased in a graded manner. In Model 3, using Q1 as the reference group, the adjusted ORs for Q2, Q3, and Q4 were 1.203 (95% CI: 1.064–1.361), 1.602 (95% CI: 1.416–1.814), and 2.559 (95% CI: 2.247–2.916), respectively.

To further address potential residual confounding related to metabolic and inflammatory status, we constructed an expanded adjustment model that additionally included lactate, WBC count, and glucose. The association between LDH and SA‐AKI remained significant in Model 4. Each 100 U/L increase in LDH was associated with increased odds of SA‐AKI (OR: 1.033, 95% CI: 1.025–1.042, *p* < 0.001). Compared with Q1, the adjusted ORs for Q2, Q3, and Q4 were 1.194 (95% CI: 1.055–1.350, *p* = 0.005), 1.585 (95% CI: 1.399–1.795, *p* < 0.001), and 2.466 (95% CI: 2.158–2.816, *p* < 0.001), respectively.

### 3.3. Sensitivity Analyses

To evaluate whether the missing data strategy materially affected the main findings, we performed a complete‐case sensitivity analysis among 6924 patients with complete data for all covariates included in the expanded Model 4. Models 1–4 were refitted within this complete‐case cohort. The results were generally consistent with those obtained using multiple imputation. Higher LDH levels remained significantly associated with SA‐AKI across models. In the expanded Model 4, each 100 U/L increase in LDH was associated with increased odds of SA‐AKI (OR: 1.045, 95% CI: 1.034–1.057, *p* < 0.001). A graded association was also observed across LDH quartiles, with adjusted ORs of 1.168, 1.519, and 2.541 for Q2, Q3, and Q4, respectively, compared with Q1 (Supporting Table S3).

As an additional sensitivity analysis, we excluded patients who fulfilled KDIGO criteria for AKI within the first 24 h of ICU admission to reduce the potential influence of temporal overlap between LDH measurement and SA‐AKI ascertainment. After excluding 3821 patients with early AKI and 288 patients with missing AKI diagnosis time, 5352 patients were included in this sensitivity analysis, among whom 1153 developed delayed SA‐AKI between 24 h and 7 days after ICU admission. The incidence of delayed SA‐AKI increased across LDH quartiles, from 14.8% in Q1 to 31.4% in Q4. In the adjusted Model 3, each 100 U/L increase in LDH remained significantly associated with delayed SA‐AKI (OR: 1.017, 95% CI: 1.008–1.026, *p* < 0.001). Compared with Q1, the adjusted ORs for Q2, Q3, and Q4 were 1.281, 1.990, and 2.717, respectively. Similar results were observed in the expanded Model 4 after additional adjustment for lactate, WBC count, and glucose (Supporting Table S4). These findings suggest that the observed association was not solely driven by patients with early or already established AKI.

### 3.4. Dose–Response Relationship Between LDH and AKI

**FIGURE 2:**
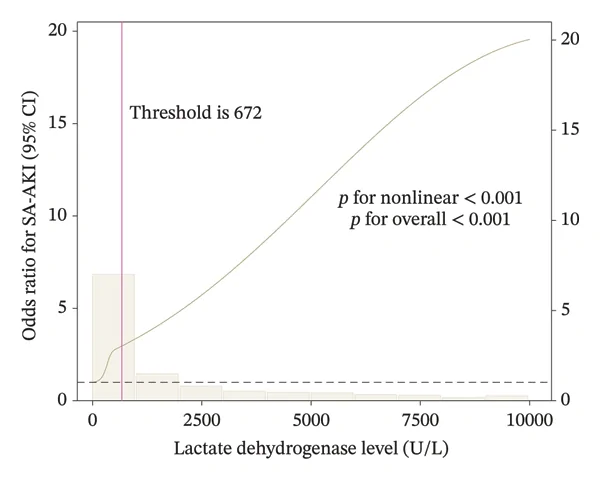
Restricted cubic spline (RCS) analysis for LDH and SA‐AKI. The curve indicates a nonlinear positive relationship between LDH and SA‐AKI odds, with the shaded band showing the 95% confidence interval. The overall and nonlinear tests were significant (both *p* < 0.001). The vertical magenta line indicates an approximate inflection point of 672 U/L, after which the estimated risk increased more rapidly.

RCS analysis demonstrated a significant nonlinear positive association between LDH levels and the risk of SA‐AKI (*p* for overall < 0.001; *p* for nonlinear < 0.001; Figure 2). The risk increased gradually at lower LDH levels and rose more sharply when LDH exceeded approximately 672 U/L, suggesting a potential threshold effect. In the present cohort, 1334 patients (14.1%) had LDH values greater than 672 U/L, indicating that this apparent threshold was not confined to a very small extreme subgroup. Overall, these findings support a nonlinear association between higher LDH levels and observed SA‐AKI risk in this cohort.

### 3.5. Subgroup Analyses

**FIGURE 3:**
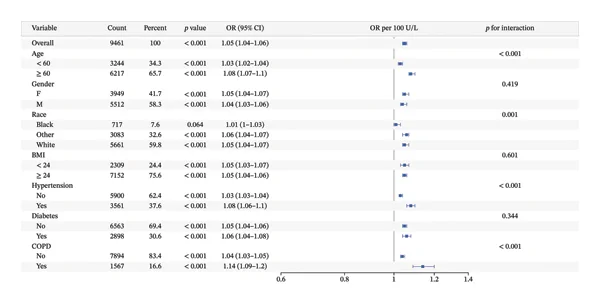
Subgroup analysis for LDH and SA‐AKI. The forest plot presents odds ratios (ORs) and 95% confidence intervals for LDH, scaled per 100 U/L, across subgroups defined by age, gender, race, BMI, hypertension, diabetes, and COPD. The direction of association was generally similar across most strata. Nominal interaction signals were seen for age, race, hypertension, and COPD (*p* for interaction < 0.05).

Subgroup analyses were performed as exploratory analyses to assess whether the association between LDH and SA‐AKI was generally consistent across clinically relevant subgroups (Figure 3). The positive association between LDH and SA‐AKI was generally observed across most subgroups, with an overall OR of 1.05 per 100 U/L increase (95% CI: 1.04–1.06; *p* < 0.001). Several interaction tests reached nominal statistical significance, including age, race, HTN, and COPD. However, these subgroup findings should be considered exploratory and interpreted cautiously, given the risks of multiple comparisons, limited power within strata, and chance findings. No significant interactions were found for gender, BMI, or diabetes status although the direction of effect was generally consistent across all subgroups.

## 4. Discussion

Using a large MIMIC‐IV cohort, we examined the relationship between serum LDH measured during the first 24 h after ICU admission and SA‐AKI. Higher LDH values corresponded to progressively higher SA‐AKI occurrence. The association persisted after adjustment for demographics, comorbidities, SOFA score, and selected metabolic and inflammatory laboratory variables. The complete‐case and expanded adjustment analyses yielded similar findings, supporting the stability of the main estimates. After patients with AKI during the first 24 h after ICU admission were excluded, LDH remained associated with delayed SA‐AKI, indicating that the findings were not driven entirely by early or established AKI. The spline analysis suggested nonlinearity, with a more pronounced increase in risk above approximately 672 U/L. Exploratory subgroup analyses showed generally similar directions of association across several strata. However, subgroup findings and additional outcomes such as renal replacement therapy and short‐term mortality were exploratory or descriptive and should not be interpreted as formal evidence of effect heterogeneity or prognostic value.

Any mechanistic interpretation should be treated as biological context and hypothesis generation rather than direct evidence from this MIMIC‐IV analysis. As a glycolytic enzyme, LDH may act as a nonspecific indicator of metabolic stress, inflammation, and hypoxia during sepsis [17]. Experimental studies link septic kidney injury to metabolic reprogramming, lactate accumulation, and lactylation‐related processes that may promote tubular injury and inflammatory amplification [18–20]. These external observations, together with our previous experimental work, offer biological plausibility for the clinical association observed here [21, 22]. Nevertheless, the present dataset did not measure these signaling or metabolic pathways, so mechanistic conclusions cannot be drawn.

Clinically, LDH is an inexpensive and widely available laboratory test in critically ill patients. In our cohort, higher LDH during the first 24 h after ICU admission corresponded to a higher observed frequency of SA‐AKI, which may reflect greater illness burden among septic ICU patients. This evidence is insufficient to establish LDH as a validated predictive marker, because the study assessed association and did not develop or validate a prediction model. Accordingly, LDH should be considered alongside other clinical information rather than used in isolation for SA‐AKI risk assessment.

Several limitations should be noted. First, the study was retrospective and based on a single database, and LDH was captured only once during the first 24 h after ICU admission. Although the temporal‐overlap sensitivity analysis reduced the influence of early or established AKI, it could not fully resolve the sequence between LDH assessment and SA‐AKI occurrence; causal inference is, therefore, not possible. Second, residual confounding may persist despite multivariable adjustment and sensitivity analyses. LDH is nonspecific and can be affected by hemolysis, liver dysfunction, skeletal muscle injury, shock severity, medications, infection site, fluid management, vasopressor therapy, mechanical ventilation, and nephrotoxic exposure, with several of these factors incompletely captured in MIMIC‐IV. Third, excluding patients without LDH measurements or complete SA‐AKI ascertainment data may have introduced selection bias. Although the imputed and complete‐case analyses were broadly consistent, bias from missing data and cohort exclusion cannot be ruled out. Fourth, because MIMIC‐IV largely reflects Western ICU practice, external validation is needed before applying the findings to other populations. Finally, subgroup analyses and descriptive outcomes were exploratory and should not be viewed as formal evidence of heterogeneity or prognostic utility.

## 5. Conclusions

In this MIMIC‐IV cohort, serum LDH measured during the first 24 h after ICU admission was nonlinearly associated with SA‐AKI. The observational design requires cautious interpretation and does not support causal inference. Prospective studies are needed to confirm the association and determine whether LDH has a clinical value for SA‐AKI risk assessment.

## Author Contributions

Nu Zhang and Zhiyu Wu performed data extraction and statistical analysis and prepared the initial manuscript draft. Mengyuan Luo contributed to data processing, figure preparation, and literature review. Zhihua Mi contributed to study design, methodological supervision, and critical manuscript revision. Qiqing Shi conceived the study, supervised the research process, interpreted the results, and finalized the manuscript for submission.

## Funding

This study received support from the Scientific Research Startup Fund of Shanghai Fourth People’s Hospital, Tongji University (Sykyqd03001), and the Discipline Advancement Program of Shanghai Fourth People’s Hospital, Tongji University (SY‐XKZT‐2022‐1012).

## Disclosure

All authors have reviewed and approved the final manuscript. The funders were not involved in the study design, data collection, analysis, interpretation, manuscript preparation, or submission decision.

## Ethics Statement

MIMIC‐IV consists of deidentified patient data and can be accessed by credentialed PhysioNet users. The Institutional Review Board at Beth Israel Deaconess Medical Center reviewed the creation of this research resource, approved the data‐sharing initiative, and waived informed consent. Accordingly, this secondary analysis did not require additional ethical approval or informed consent.

## Consent

Not applicable because only deidentified MIMIC‐IV data were used.

## Conflicts of Interest

The authors declare no conflicts of interest.

## Supporting Information

Additional supporting information can be found online in the Supporting Information section.

The Supporting Information contains the following items:

## Supporting information


**Supporting Information 1** Supporting Figure S1: Directed acyclic graph describing the assumed relationships among demographics, comorbidities, illness severity, metabolic and inflammatory status, serum LDH, and SA‐AKI.

**Supporting Information 2** Supporting Table S1: Missingness of candidate covariates before multiple imputation.

**Supporting Information 3** Supporting Table S2: Comparison of baseline characteristics between patients included in the final analytic cohort and patients excluded because of missing LDH measurements or incomplete SA‐AKI ascertainment data.

**Supporting Information 4** Supporting Table S3: Complete‐case sensitivity analysis for the association between LDH and SA‐AKI.

**Supporting Information 5** Supporting Table S4: Sensitivity analysis for delayed SA‐AKI after excluding patients with AKI during the first 24 h after ICU admission.

## Data Availability

The data used for this analysis came from MIMIC‐IV Version 3.1, a publicly accessible critical care database hosted by PhysioNet. Access is limited to credentialed users who complete the required training and data use agreement. Eligible researchers can request access directly through PhysioNet.
