# Supplementary material for: Association of Serum Lactate Dehydrogenase With Sepsis‐Associated Acute Kidney Injury: A Large‐Scale Analysis Based on the MIMIC‐IV Database
Source: Emerg Med Int. 2026 Aug 24;2026:9179449. doi: 10.1155/emmi/9179449 (PMC13501253; doi:10.1155/emmi/9179449)
Supplement: Supplementary file 1 [file EMMI-2026-9179449-s001.docx]

**Supplementary Figure S1. Directed acyclic graph illustrating covariate selection.** Demographic factors, comorbidities, illness severity, and metabolic/inflammatory status were considered potential confounders because they may influence both LDH levels and the risk of SA-AKI. Serum creatinine and urine output were not included in the expanded adjustment model because they were directly involved in KDIGO-based SA-AKI ascertainment. CRRT and mortality were considered downstream clinical outcomes and were therefore not adjusted for in the primary association models.
