# Supplementary material for: Association of Serum Lactate Dehydrogenase With Sepsis‐Associated Acute Kidney Injury: A Large‐Scale Analysis Based on the MIMIC‐IV Database
Source: Emerg Med Int. 2026 Aug 24;2026:9179449. doi: 10.1155/emmi/9179449 (PMC13501253; doi:10.1155/emmi/9179449)
Supplement: Supplementary file 2 [file EMMI-2026-9179449-s002.docx]

### Supplementary Table S1. Variable-level missingness among candidate covariates in the analytic cohort

| **Variable** | **Missing n** | **Missing %** | **Handling in analysis** |
| --- | --- | --- | --- |
| Age | 0 | 0.00% | Included directly |
| Sex | 0 | 0.00% | Included directly |
| BMI | 2244 | 23.72% | Multiple imputation (BMI derived from weight and height) |
| Race | 0 | 0.00% | Included directly |
| SOFA score | 0 | 0.00% | Included directly |
| Creatinine | 2 | 0.02% | Multiple imputation for descriptive analyses; not included in Model 4 |
| Lactate | 562 | 5.94% | Multiple imputation |
| Glucose | 3 | 0.03% | Multiple imputation |
| White blood cell count | 44 | 0.47% | Multiple imputation |
| Hypertension | 0 | 0.00% | Included directly |
| Diabetes mellitus | 0 | 0.00% | Included directly |
| COPD | 0 | 0.00% | Included directly |

**Notes:** This table summarizes variable-level missingness before multiple imputation among patients included in the final analytic cohort. Variables with more than 25% missingness were excluded from the adjusted models. Variables with 25% or less missingness were imputed using the multiple imputation module implemented in DecisionChain software. Serum creatinine was summarized descriptively but was not included in the expanded adjustment model because it was directly involved in KDIGO-based SA-AKI ascertainment.
