# Supplementary material for: Association of Serum Lactate Dehydrogenase With Sepsis‐Associated Acute Kidney Injury: A Large‐Scale Analysis Based on the MIMIC‐IV Database
Source: Emerg Med Int. 2026 Aug 24;2026:9179449. doi: 10.1155/emmi/9179449 (PMC13501253; doi:10.1155/emmi/9179449)
Supplement: Supplementary file 3 [file EMMI-2026-9179449-s003.docx]

**Supplementary Table S2. Comparison of baseline characteristics between included and excluded patients**

Included patients were those included in the final analytic cohort (N = 9,461). Excluded patients were those excluded because of missing LDH measurements within 24 hours of ICU admission or incomplete information required for SA-AKI ascertainment (N = 8,965).

| **Variable** | **Included patients (N = 9,461)** | **Excluded patients (N = 8,965)** | **P value** |
| --- | --- | --- | --- |
| Age, years | 66.00 (55.00-77.00), n=9,461 | 69.00 (58.00-80.00), n=8,965 | <0.001 |
| Male sex | 5,512 (58.3%), n=9,461 | 5,089 (56.8%), n=8,965 | 0.040 |
| **Race** |  |  | **<0.001** |
| White | 5,661 (59.8%), n=9,461 | 6,026 (67.2%), n=8,965 |  |
| Black | 868 (9.2%), n=9,461 | 680 (7.6%), n=8,965 |  |
| Other | 2,932 (31.0%), n=9,461 | 2,259 (25.2%), n=8,965 |  |
| BMI, kg/m2 | 28.23 (24.29-33.36), n=7,217 | 28.11 (24.44-33.09), n=5,489 | 0.415 |
| SOFA score | 7.00 (4.00-10.00), n=9,461 | 5.00 (3.00-7.00), n=8,965 | <0.001 |
| Hypertension | 3,561 (37.6%), n=9,461 | 3,949 (44.0%), n=8,965 | <0.001 |
| Diabetes mellitus | 2,898 (30.6%), n=9,461 | 2,672 (29.8%), n=8,965 | 0.222 |
| COPD | 1,567 (16.6%), n=9,461 | 1,383 (15.4%), n=8,965 | 0.036 |
| ICU length of stay, days | 7.15 (4.60-12.81), n=9,461 | 2.99 (2.40-5.05), n=8,965 | <0.001 |
| Hospital length of stay, days | 15.53 (9.27-25.25), n=9,461 | 8.83 (5.81-14.45), n=8,965 | <0.001 |
| 28-day ICU mortality | 2,145 (22.7%), n=9,461 | 1,182 (13.2%), n=8,965 | <0.001 |
| 28-day in-hospital mortality | 2,048 (21.6%), n=9,461 | 1,173 (13.1%), n=8,965 | <0.001 |

Notes: Data are presented as median (interquartile range [IQR]) or n (%). Comparisons were performed using the Mann-Whitney U test for continuous variables and the chi-square test or Fisher's exact test for categorical variables, as appropriate. The number of available observations is reported for each variable where applicable. BMI was calculated from available height and weight data. LDH, SA-AKI, and CRRT were not compared because these variables were part of the exclusion criteria, outcome definition, or downstream renal outcomes.

Abbreviations: BMI, body mass index; SOFA, Sequential Organ Failure Assessment; COPD, chronic obstructive pulmonary disease; ICU, intensive care unit; LDH, lactate dehydrogenase; SA-AKI, sepsis-associated acute kidney injury; CRRT, continuous renal replacement therapy.
