# Supplementary material for: Association of Serum Lactate Dehydrogenase With Sepsis‐Associated Acute Kidney Injury: A Large‐Scale Analysis Based on the MIMIC‐IV Database
Source: Emerg Med Int. 2026 Aug 24;2026:9179449. doi: 10.1155/emmi/9179449 (PMC13501253; doi:10.1155/emmi/9179449)
Supplement: Supplementary file 4 [file EMMI-2026-9179449-s004.docx]

**Supplementary Table S3. Complete-case sensitivity analysis of the association between serum LDH and SA-AKI**

All models were refitted in the same complete-case cohort with complete data for all covariates included in the expanded Model 4 (n = 6,924; SA-AKI events = 4,059).

| **LDH modeling** | **LDH category / scale** | **Model 1 OR (95% CI)** | **Model 1 P value** | **Model 2 OR (95% CI)** | **Model 2 P value** | **Model 3 OR (95% CI)** | **Model 3 P value** | **Model 4 OR (95% CI)** | **Model 4 P value** |
| --- | --- | --- | --- | --- | --- | --- | --- | --- | --- |
| Continuous LDH | Per 100 U/L increase | 1.061 (1.049–1.073) | <0.001 | 1.066 (1.054–1.079) | <0.001 | 1.048 (1.036–1.060) | <0.001 | 1.045 (1.034–1.057) | <0.001 |
| LDH quartiles | Q1 (≤229 U/L) | Reference |  | Reference |  | Reference |  | Reference |  |
|  | Q2 (230–313 U/L) | 1.340 (1.167–1.538) | <0.001 | 1.272 (1.104–1.465) | <0.001 | 1.183 (1.019–1.373) | 0.028 | 1.168 (1.006–1.357) | 0.041 |
|  | Q3 (314–478 U/L) | 1.810 (1.577–2.078) | <0.001 | 1.782 (1.546–2.054) | <0.001 | 1.564 (1.347–1.816) | <0.001 | 1.519 (1.307–1.765) | <0.001 |
|  | Q4 (>478 U/L) | 3.138 (2.724–3.616) | <0.001 | 3.331 (2.876–3.858) | <0.001 | 2.655 (2.275–3.100) | <0.001 | 2.541 (2.170–2.975) | <0.001 |

Abbreviations: CI, confidence interval; COPD, chronic obstructive pulmonary disease; DM, diabetes mellitus; HTN, hypertension; LDH, lactate dehydrogenase; OR, odds ratio; SA-AKI, sepsis-associated acute kidney injury; SOFA, Sequential Organ Failure Assessment; WBC, white blood cell.

Model 1: unadjusted.

Model 2: adjusted for age, gender, BMI, and race.

Model 3: adjusted for age, gender, BMI, race, SOFA score, HTN, DM, and COPD.

Model 4: adjusted for age, gender, BMI, race, SOFA score, HTN, DM, COPD, lactate, WBC count, and glucose.

LDH quartiles were defined using the cutoffs from the primary analytic cohort: Q1 ≤229 U/L, Q2 230–313 U/L, Q3 314–478 U/L, and Q4 >478 U/L.

ORs were estimated using logistic regression with SA-AKI as the dependent variable. Continuous LDH was scaled per 100 U/L increase.
