# Supplementary material for: Association of Serum Lactate Dehydrogenase With Sepsis‐Associated Acute Kidney Injury: A Large‐Scale Analysis Based on the MIMIC‐IV Database
Source: Emerg Med Int. 2026 Aug 24;2026:9179449. doi: 10.1155/emmi/9179449 (PMC13501253; doi:10.1155/emmi/9179449)
Supplement: Supplementary file 5 [file EMMI-2026-9179449-s005.docx]

**Supplementary Table S4. Sensitivity analysis after excluding patients with early AKI within 24 hours of ICU admission**

**A. Patient classification for temporal sensitivity analysis**

| **Category** | **No. of patients** | **Definition / notes** |
| --- | --- | --- |
| Original study cohort | 9,461 | Final cohort included in the primary analysis |
| Patients with SA-AKI in the primary cohort | 5,317 | Patients coded as AKI = 1 |
| Patients without SA-AKI in the primary cohort | 4,144 | Patients coded as AKI = 0 |
| Excluded: early AKI within 24 hours after ICU admission | 3,821 | AKI diagnosed ≤24 hours after ICU admission |
| Excluded: missing AKI diagnosis time | 288 | AKI = 1 but missing first AKI diagnosis time |
| AKI first diagnosed >7 days after ICU admission | 55 | AKI first diagnosed >7 days after ICU admission; not counted as delayed SA-AKI within 7 days. |
| Included in temporal sensitivity analysis | 5,352 | Original cohort minus early AKI and missing AKI diagnosis time |
| Delayed SA-AKI events | 1,153 | AKI first diagnosed >24 hours and ≤7 days after ICU admission |
| Non-events in temporal sensitivity analysis | 4,199 | No delayed SA-AKI within 7 days after ICU admission |

AKI, acute kidney injury; ICU, intensive care unit; SA-AKI, sepsis-associated acute kidney injury.

**B. Incidence of delayed SA-AKI across LDH quartiles after excluding early AKI cases**

| **LDH quartile** | **N** | **Delayed SA-AKI** | **Non-event** | **Incidence** |
| --- | --- | --- | --- | --- |
| Q1 | 1,577 | 234 | 1,343 | 14.8% |
| Q2 | 1,423 | 259 | 1,164 | 18.2% |
| Q3 | 1,312 | 333 | 979 | 25.4% |
| Q4 | 1,040 | 327 | 713 | 31.4% |

Delayed SA-AKI was defined as AKI first diagnosed >24 hours and ≤7 days after ICU admission.

**C. Association between LDH and delayed SA-AKI after excluding early AKI cases**

| **Exposure** | **Model 1 OR (95% CI)** | **P value** | **Model 2 OR (95% CI)** | **P value** | **Model 3 OR (95% CI)** | **P value** | **Model 4 OR (95% CI)** | **P value** |
| --- | --- | --- | --- | --- | --- | --- | --- | --- |
| Continuous LDH, per 100 U/L increase | 1.017 (1.009–1.026) | <0.001 | 1.020 (1.011–1.029) | <0.001 | 1.017 (1.008–1.026) | <0.001 | 1.019 (1.009–1.028) | <0.001 |
| **LDH quartiles** |  |  |  |  |  |  |  |  |
| Q1 (reference) | 1 |  | 1 |  | 1 |  | 1 |  |
| Q2 | 1.277 (1.052–1.550) | 0.013 | 1.265 (1.041–1.537) | 0.018 | 1.281 (1.053–1.559) | 0.013 | 1.307 (1.073–1.592) | 0.008 |
| Q3 | 1.952 (1.620–2.352) | <0.001 | 2.040 (1.690–2.463) | <0.001 | 1.990 (1.645–2.407) | <0.001 | 2.042 (1.686–2.473) | <0.001 |
| Q4 | 2.632 (2.175–3.186) | <0.001 | 2.831 (2.331–3.437) | <0.001 | 2.717 (2.232–3.309) | <0.001 | 2.884 (2.358–3.527) | <0.001 |

Model 1: unadjusted model. Model 2: adjusted for age, gender, BMI, and race. Model 3: further adjusted for SOFA score and comorbidities including hypertension, diabetes mellitus, and COPD. Model 4: further adjusted for lactate, white blood cell count, and glucose. LDH quartiles were defined according to the primary analysis: Q1, ≤229 U/L; Q2, 230–313 U/L; Q3, 314–478 U/L; and Q4, >478 U/L. LDH, lactate dehydrogenase; OR, odds ratio; CI, confidence interval; BMI, body mass index; SOFA, Sequential Organ Failure Assessment; COPD, chronic obstructive pulmonary disease.
